# microRNA in Cardiovascular Aging and Age-Related Cardiovascular Diseases

**DOI:** 10.3389/fmed.2017.00074

**Published:** 2017-06-12

**Authors:** Claudio de Lucia, Klara Komici, Giulia Borghetti, Grazia Daniela Femminella, Leonardo Bencivenga, Alessandro Cannavo, Graziamaria Corbi, Nicola Ferrara, Steven R. Houser, Walter J. Koch, Giuseppe Rengo

**Affiliations:** ^1^Division of Geriatrics, Department of Translational Medical Sciences, Federico II University of Naples, Naples, Italy; ^2^Center for Translational Medicine, Department of Pharmacology, Lewis Katz School of Medicine, Temple University, Philadelphia, PA, United States; ^3^Cardiovascular Research Center, Lewis Katz School of Medicine, Temple University, Philadelphia, PA, United States; ^4^Department of Medicine and Health Sciences, University of Molise, Campobasso, Italy; ^5^Scientific Institute of Telese Terme, Salvatore Maugeri Foundation, IRCCS, Benevento, Italy

**Keywords:** microRNA, aging, cardiovascular diseases, heart failure, atherosclerosis, hypertension, diabetes, atrial fibrillation

## Abstract

Over the last decades, life expectancy has significantly increased although several chronic diseases persist in the population, with aging as the leading risk factor. Despite improvements in diagnosis and treatment, many elderlies suffer from cardiovascular problems that are much more frequent in an older, more fragile organism. In the long term, age-related cardiovascular diseases (CVDs) contribute to the decline of quality of life and ability to perform normal activities of daily living. microRNAs (miRNAs) are a class of small non-coding RNAs that regulate gene expression at the posttranscriptional level in both physiological and pathological conditions. In this review, we will focus on the role of miRNAs in aging and age-related CVDs as heart failure, hypertension, atherosclerosis, atrial fibrillation, and diabetes mellitus. miRNAs are key regulators of complex biological mechanisms, representing an exciting potential therapeutic target in CVDs. Moreover, one major challenge in geriatric medicine is to find reliable biomarkers for diagnosis, prognosis, and prediction of the response to specific drugs. miRNAs represent a very promising tool due to their stability in the circulation and unique signature in CVDs. However, further studies are needed to investigate their translational potential in the real clinical practice.

## Introduction

Cardiovascular diseases (CVDs) in older patients inflict a huge burden in terms of mortality and morbidity, contributing to disability and decline of quality of life (1–3). In addition, aging itself produces numerous changes in human heart, at structural, molecular, and functional levels. The most important age-related alterations are left ventricular (LV) hypertrophy, fibrosis, denervation, and maladaptive remodeling that frequently lead to diastolic dysfunction (4–7). Vascular aging is characterized by impaired endothelial function, chronic vascular inflammation, and augmented arterial stiffness (8). Endothelial dysfunction is mainly related to increased production of reactive oxygen species (ROS) partially due to an increased activity of NAD(P)H oxidases (NOX) (9). Moreover, increased age-related oxidative stress and decreased production of nitric oxide (NO) constitute critical factors for the alteration of cardiovascular (CV) homeostasis (10). microRNAs (miRNAs) are small non-coding RNAs approximately 18–25 nucleotides long, which regulate gene expression at different levels. Importantly, miRNAs are emerging as key factors in the development and progression of CVDs. Notably several basic and clinical studies identified peculiar miRNAs expression profile in pathological conditions. Consequently, miRNA have been proposed as diagnostic/prognostic biomarkers and therapeutic targets.

The present article will focus on the regulation and function of miRNAs in CV aging and age-related CVDs as heart failure (HF), hypertension, atherosclerosis, atrial fibrillation (AF), and diabetes mellitus (DM).

## microRNAs

### Biogenesis and Nomenclature

**Figure 1:**
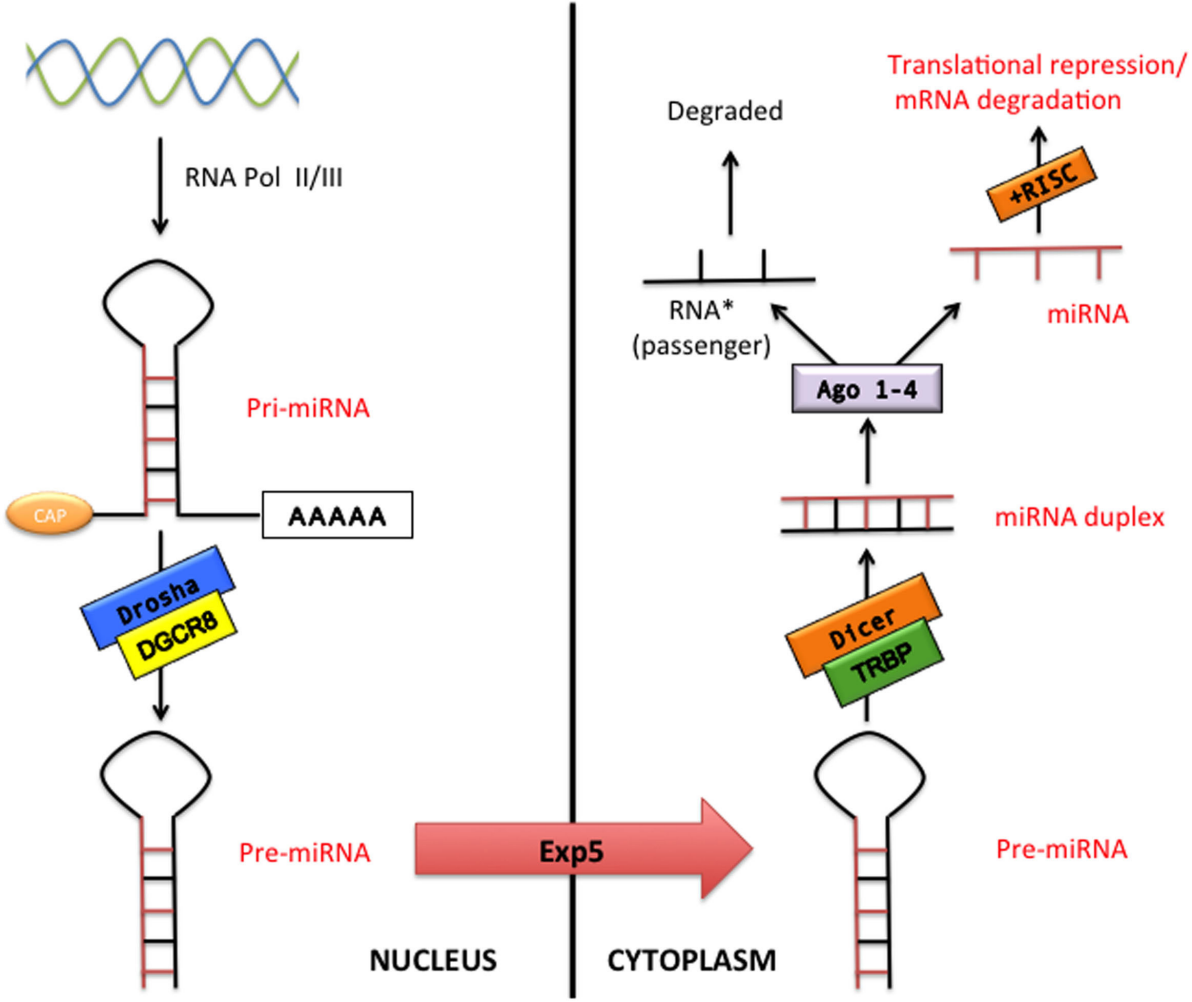
The canonical microRNA (miRNA) pathway. pri-miRNA are transcribed by RNA polymerase II or III (RNA pol II/III) and then processed by Drosha/DiGeorge syndrome critical region gene 8 (DGCR8) into pre-miRNA transcripts. Pre-miRNAs are exported from the nucleus *via* exportin-5 (Exp 5) to cytoplasm where processed by Dicer/TAR RNA-binding protein (TRBP), which generates double-stranded RNAs (miRNA duplex). Argonaute proteins 1–4 (Ago 1–4) unwind and then separate the guide strand (miRNA) and the passenger strand (miRNA passenger). The RNA-induced silencing complex (RISC) incorporates the mature miRNA and regulates gene expression by translation repression or mRNA degradation.

microRNAs constitute a class of small non-coding RNAs that regulate gene expression at the posttranscriptional level in both physiological and pathological conditions. miRNAs are highly conserved during evolution with a well-established role in cell proliferation, differentiation, metabolism, apoptosis, development, and aging as well as in the pathophysiology of many diseases such as cancer, CVDs, and neurological disorders (11). The miRNA coding regions are mainly present in the genome as clusters (12). The classical miRNA production is called “the canonical pathway” (Figure 1). Essentially, miRNAs are transcribed in the nucleus by the RNA polymerase II (or in few cases RNA polymerase III) as a pri-miRNA. pri-miRNA represents a primary long transcript (>1 kb) bearing a hairpin-shaped structure that provisionally displays a 5′-Cap and a 3′-poly(A) tail (13–15). pri-miRNAs are then cleaved into around 70-nucleotide pre-miRNAs by the microprocessor complex that contains Drosha (a RNAse III enzyme) and its cofactor DiGeorge syndrome critical region gene 8 (DGCR8) (13, 16). The pre-miRNAs are subsequently exported to the cytoplasm by exportin-5, *via* a RAN-GTP-dependent mechanism, where they are processed into 20–25 nucleotide double-stranded-RNAs (miRNA duplex) by Dicer (another RNAse III enzyme) and by its cofactor TAR RNA-binding protein (13). In the cytoplasm, miRNA duplex is finally separated into two RNA strands, the guide RNA strand (miRNA) and the passenger RNA strand (miRNA passenger) that is generally degraded. The miRNA is then bound by Argonaute proteins 1–4 and loaded into the RNA-induced silencing complex in order to influence the target mRNA expression (13). The miRNA target sites, known as miRNA recognition elements, are frequently located in the 3′-untranslated region of the mRNA. It has been also described that miRNAs can bind to the 5′-untranslated region or to the open reading frame (13, 17, 18). miRNAs can repress gene target by either mRNA decay or translation repression (19). Interestingly, it has been reported that some miRNA bypass particular maturation steps of the canonical miRNA pathway and follow an alternative miRNA biogenesis (13): some authors showed the biogenesis of specific miRNA to be Dicer-independent, while other miRNAs do not need Drosha/DGCR8 for their maturation (so-called Mirtron pathway) (13). Recently, a third pathway (Agotron pathway) escapes both Drosha and Dicer processing and seems to limit off-target effects (13). The nomenclature of miRNA is indicated into the miRNA register and identifying numbers have been assigned serially (20–23).

Identical miRNAs have the same number, regardless of the specie. The pre-miRNA is referred as “mir-,” while a capitalized “miR-” suggests the mature form. The “mir” or “miR” are followed by a dash and a number (e.g., mir-22 or miR-22). Pre-miRNAs producing an identical mature miRNAs but positioned at different places in the genome are labeled with an additional dash-number suffix (e.g., mir-33-1 and mir-33-2 lead to an identical mature miRNA, miR-33). Different mature miRNAs with almost identical sequences except for one or two nucleotides are marked with an additional lower case letter (e.g., miR-29a is strictly related to miR-29b). Two different mature miRNA strands that are cleaved from opposite arms of the same miRNA duplex have been conventionally designated for example as miR-75-5p (5′ arm) and miR-75-3p (3′ arm). However, relative expression levels are frequently known and an asterisk following the name indicates an miRNA expressed at low levels relative to the one in the opposite arm of a hairpin (low expressed miRNA has been previously indicated as RNA passenger): for example, miR-21 and miR-21* (20–24).

### Biomarkers and Therapeutic Options

The possibility to detect different miRNAs in numerous body fluids (blood, saliva, urine, etc.) opens up the opportunity to use these molecules as biomarker for age-related CVDs. Importantly, miRNAs are simply quantified by real-time polymerase chain reaction or microarrays and a combination of multiple miRNAs have been shown to provide a more sensitive and specific information (25–27). Serum and plasma miRNAs are probably the most suitable source for clinical application as sample collection is quick, simple, and reproducible. Indeed, miRNAs are remarkably stable and resistant in the blood and long-term storage or freezing/thawing cycles do not seem to cause problems (28, 29). Interestingly, circulating miRNAs are protected from endogenous high levels of RNase activity in the blood since they are linked to carriers in vesicles or non-vesicular forms (30). The non-vesicular forms account for 80–99% of total circulating miRNAs, which are linked to RNA-binding proteins in very stable complexes (Ago2-family proteins or nuclephosmine 1), or to lipoproteins as high-density lipoproteins (HDLs) (31–34). In addition, miRNAs can be integrated into extracellular vesicles such as exosomes, microvesicles, and apoptotic bodies (34). These microvesicles are secreted from the cell and involved in the communication between cells through transfer of genetic information. Exosomes (30–150 nm) are produced *via* inward budding of endosomal membranes that merge with the plasma membrane and then release their content into the extracellular environment as vesicles (34–37). On the contrary, microvesicles are larger (50–1,000 nm) vesicles generated by outward budding and fission from the cell surface, while apoptotic bodies (50 nm to 5 µm) result from cells during the late stages of apoptosis (34, 38, 39). Of note, Ago2 influences the function of secreted miRNAs in origin cell-secreted microvesicles (but not in recipient cells) (40).

In the last decade, miRNA-based therapeutics are emerging as promising and effective treatments.

Compared to current drugs, miRNA-based therapies target several genes that are under the control of the specific miRNA. miRNAs can be manipulated through replacement by specific oligonucleotides (miR-mimics) or inhibited by single-stranded antisense oligonucleotides (antimiRs or antagomirs) (24, 41). The miR-mimics are used in case of disease-related downregulation of a specific miRNA and are usually synthetized as double-stranded miRNA oligonucleotides that, when transfected into cells, are processed into a single-strand form and regulate target genes (42, 43). On the contrary, antagomiRs are modified antisense oligonucleotides that can inhibit the levels of upregulated miRNAs, thus increasing the expression of mRNAs targeted by that specific miRNA (42–44). The proposed routes of *in vivo* delivery of miRNA therapeutics to target CV system are intravenous, subcutaneous, intramuscular, intraperitoneal, cardiac, or transcoronary but it is still unclear which one among them is the most effective. However, since local delivery is particularly desirable for the miRNA-based treatment, cardiac or intracoronary injections probably represent an optimal choice in case of heart disease (HF due to different etiologies or age-related heart dysfunction) (41, 45, 46). The main delivery strategies/technologies for miRNA therapeutics include lipid-based vehicles, viral systems, biodegradable scaffolds, “exosome-encapsulated” miRNAs, or light-induced antagomir activation (46–48).

## miRNAs in Cardiovascular Aging

### miRNAs in Cardiac Aging

Physiological cardiac aging is associated with an increase in cardiac fibrosis, LV hypertrophy, valvular degeneration, and mainly diastolic dysfunction (4).

Numerous miRNAs have been described to be differently expressed and to regulate different cell types and pathways during cardiac aging (see Table 1) (49, 50). Zhang et al. showed that cardiac miR-21 is upregulated with age (50). miR-21 profibrotic effect is induced *via* ERK–MAP kinase pathway activation in cardiac fibroblasts (CFs) during injury (see below) (51).

**Table 1:** Summarizing table of microRNAs (miRNAs) involved in cardiac and vascular aging.

Tissue	miRNA	Molecular targets	Functions	Reference
Aging heart	miR-21 ↑	ERK–MAP kinase signaling	Profibrotic [role on cardiac fibroblast (CFs)]	(51)
Aging heart	miR-22 ↑	Mimecan/Osteoglycin	Accelerate CF senescence and migration	(52)
Aging heart	miR-18 and miR-19 ↓	Thrombospondin-1 and connective tissue growth factor	Anti-heart failure-related fibrosis during aging	(58)
Aging heart	miR-17-3p ↓	PAR-4	Reduce CF cellular senescence	(61)
Aging heart	miR-34a ↑	Phosphatase 1 nuclear targeting subunit	Increase age-related cardiomyocyte apoptosis and cardiac dysfunction	(62)
Vascular aging	miR-34a ↑	SIRT1	Stimulates senescence in endothelial cell and vascular smooth muscle cells	(68, 71)
Vascular aging	miR-217 ↑	SIRT1	Stimulates endothelial senescence decreasing nitric oxide	(73)
Vascular aging	miR-29 ↑	Collagen and elastin	Extracellular matrix impairment (risk of age-related aortic aneurism)	(74–77)
Vascular aging	miR-146 ↑ or ↓	IRAK and NOX4	Pro-inflammation or anti-oxidative stress	(78, 79)
Vascular aging	miR-92 ↓	Tumor necrosis factor α receptor 1 and collagen type 1	miR-92 reduction mimic arterial aging	(80)

In addition, overexpression of Ago1 and Ago2 synergistically induced miR-21 and miR-21*, suggesting a regulatory role for Ago proteins (50).

Another miRNA involved in cardiac aging is miR-22 (52). Age-related miR-22 upregulation contributed to accelerate CF senescence and increased migration (52). The effects of miR-22 in CFs were mediated by the proteoglycan mimecan/osteoglycin (52). miR-22 regulatory sequence deletion in Mimecan gene was able to revert in part the effects of miR-22, recommending Mimecan as a potential target (52). Interestingly, Mimecan/osteoglycin has been previously shown to regulate arteriogenesis, collagen fibrillogenesis in the extracellular matrix (ECM), and cardiac hypertrophy (see below) (53–55). Moreover, Gupta et al. have recently suggested a role for miR-22 in aged cardiomyocytes (56). The inhibition of miR-22 stimulated cardiac autophagy, curbed maladaptive remodeling, and enhanced cardiac function post-myocardial infarction (MI) in older mice, but not in younger ones (56). On the other hand, a recent integromics network meta-analysis indicates that miR-22 does not importantly impact on heart longevity when compared to other age-modified miRNAs (57). miR-17-92 cluster has been widely shown to be involved in cardiac aging and consists of six mature miRNAs: miR-17, miR-18a, miR-19a, miR-19b, miR-20a, and miR-92a. van Almen et al. found that miR-18 and miR-19 levels were decreased in an aged HF-prone mouse strain when compared to age-matched controls (58, 59). These findings were also confirmed in human samples, and this is crucial since HF is a frequent comorbidity in elderly patients (58, 60).

miR-18 and miR-19 downregulation in the heart was associated with increased levels of connective tissue growth factor (CTGF) and thrombospondin-1, fundamental in the remodeling of the ECM. Importantly, the authors demonstrated that this pathway is specific for cardiomyocytes (rather than CFs) and influences collagen production (58).

miR-17-3p has been shown to reduce cardiac aging and CF cellular senescence by targeting Par4, instead (61).

Remarkably, miR-34a expression was significantly augmented in aged human hearts, in a mouse model of accelerated aging or post-MI (62). The latter finding suggests miR-34a to constitute a promising candidate for the treatment of elderly patient suffering from postischemic HF. In fact, the inhibition of miR-34a through gene deletion or antagomiR was able to reverse both postischemic and age-related cardiac dysfunction. The authors showed anti-miR-34a therapy to reduce cardiomyocyte cell death *via* phosphatase 1 nuclear targeting subunit and regulation of telomere length (62).

### miRNAs in Vascular Aging

Vascular aging is characterized by endothelial dysfunction, increased fibrosis, and arterial stiffness (63). The wall of large arteries, particularly the aorta, is thicker and less elastic during aging, mainly due to progressive deterioration and loss of elastin together with increase in collagen fibers (64, 65). In addition, hypertension and atherosclerosis are extremely frequent in the elderly population and substantially contribute to biomechanical and structural alterations of the vascular system (see below for specific miRNA involved in pathophysiology of hypertension and atherosclerosis) (66). On the other hand, increased aortic stiffness has been recently associated with higher risk of incident hypertension (67).

Numerous studies have evaluated the role of miRNAs during aging in cell models of vascular senescence or in vessels isolated from animal models of physiological aging (Table 1).

miR-34a has been shown to be upregulated in senescent endothelial cells (ECs) and vascular smooth muscle cells (VSMCs) as well as in cultured endothelial progenitor cells and bone marrow-derived pro-angiogenic cells from elderly patients with coronary artery disease (CAD) or from mouse model of physiological aging (68–71). miR-34a main target is SIRT1, a histone deacetylase representing both a longevity-stimulating enzyme and an important component for endothelial function *via* Notch signaling (68, 72). Similar to miR-34, miR-217 has been shown to be overexpressed in human umbilical vein endothelial cells (HUVECs) and to downregulate SIRT1. In addition, miR-217 upregulation determined higher acetylation of SIRT-1 target genes like FoxO1 and endothelial nitric oxide synthase (eNOS) (73). miR-29 family members (miR-29a, miR-29b, and miR-29c) are upregulated in the aorta of aged mice and decreased levels of collagens and elastin, crucial ECM components (74, 75). Importantly, miR-29a was significantly augmented in cultured senescent ECs, suggesting their involvement in the phenomenon (75). Furthermore, an antagomiR directed to miR-29 increased ECM proteins expression in Angiotensin II (Ang II)-induced aneurysm model aged mice, as well as in genetic models of thoracic aortic aneurysm formation; this evidence even confirmed the relevance of this miRNA *in vivo* (74–77). The role of miR-146 is controversial: some authors found miR-146 to be upregulated in cellular model of endothelial senescence, otherwise another group reported its downregulatation. In the first study, miR-146 upregulation in ECs was related to reduced IRAK levels (a protein involved in the inflammatory response) while in the second one its induced overexpression favorably lowered NOX4 levels (78, 79). Consequently, *in vivo* studies are necessary to determine the role of miR-146a during vascular aging.

miR-92a is reduced both in arteries of older adults and mouse model of vascular aging (80). miR-92a inhibition (through an antagomiR) in young mice showed a phenotype similar to the one typical of arterial aging *via* tumor necrosis factor α receptor 1 and collagen type 1 upregulation. However, in order to determine the real efficacy of miR-92a *in vivo*, it is necessary to evaluate if its upregulation is able to revert age-related vascular dysfunction.

### Circulating miRNAs during Aging

microRNAs are secreted in the extracellular space and are emerging as important molecules for paracrine and systemic communication between different cells, organs, and systems. Thus, a combination of specific circulating miRNAs would be a valuable tool to estimate the age-related deterioration of different organs.

Currently, geriatricians are using numerous clinical multidimensional indexes to identify frail elderly patients and to predict the risk of mortality (81, 82). In fact, multidimensional scales usually evaluate medical comorbidities, psychosocial behavior, and functional capabilities of elderlies in order to quantify overall health during aging (83–85). Despite several studies have been performed, current multidimensional instruments are not adequate to extensively stratify the risk for hospitalization and mortality in old people since influenced by different comorbidities and factors. Indeed, a considerable limit of these frailty scales is that different tools have been shown to be only effective in specific clinical conditions or settings (e.g., HF, cancer, or dementia; population-level frailty screening versus clinical screening) and, so far, no assessment scale is applicable to every elderly patient (83, 84, 86, 87).

Therefore, to add new circulating biomarkers as circulating miRNAs to current geriatric tools would be definitely valuable. Circulating miRNAs have potential uses for the diagnosis and prognosis of many age-related CVDs; their levels have been correlated with different ages and some of them have been associated with successful aging (see below). At this regard, it has been shown that aging *per se* influences circulating levels of some miRNAs in animal models of physiological aging as well in elderly subjects compared to younger (88–90). Victoria and colleagues performed a network analysis of circulating miRNAs and found that miR-34b/c and miR-449 expression levels correlated with age (88). This finding is not surprising since miR-34 family members (miR-34a, -34b, and -34c) have already been associated with cardiac dysfunction as well as Alzheimer’s disease both in animal models and humans (91, 92). Moreover, Victoria et al. showed that plasma levels of miR-146a, already associated with endothelial senescence (see above), rises in aged normal mice, but was unchanged in dwarf mice that have increased life span (88, 89). In addition, many studies found a different circulating miRNA profile in centenarians (“extraordinary successful aging”) compared to septuagenarians (“ordinary aging”) (90, 93). Circulating levels of miR-21-5p increase during aging and strictly relate to a longevity phenotype (downregulated in successful aging compared to ordinary aging) (90, 94–96). Interestingly, cardiac miR-21 has been also shown to be increased in a mouse model of aging (Table 1) (50). However, increased levels of circulating miR-21 in aged patients would be almost partially due to its connection with MI/age-related cardiac dysfunction and cancer, frequent comorbidities in elderly (50, 97, 98). Remarkably, miR-181 was downregulated in peripheral blood of older subject and further reduced in age-matched patients with HF (99).

## miRNAs in Heart Failure

Heart failure is a clinical syndrome characterized by a decline in contractile function, activation of several neurohormonal mechanisms, and cardiac remodeling. Although initially constituting an adaptive process to maintain cardiac output, LV remodeling plays a fundamental role in the progression to HF and is characterized by cardiomyocytes hypertrophy, apoptosis, and excessive myocardial fibrosis (100). Several signaling pathways and systems, such as sympathetic nervous system (SNS), renin–angiotensin–aldosterone system (RAAS), and inflammatory pathway, involved in the pathogenesis of HF, have been widely studied (101–103). Incidence and prevalence of HF increase with aging, and exacerbation of HF is one of the leading causes of hospitalization among over 65 years older people (104). Recently, multiple publications have reported relevant implications of tissue and circulating mRNAs in cardiac function, in specific processes of cardiac remodeling and also in the prognosis of MI (summary in Table 2) (105, 106).

**Table 2:** Summarizing table of main effects of different microRNAs (miRNAs) reported in the text within specific cardiac remodeling characteristics.

miRNA	Cardiac remodeling implication	Effect	Reference
miR-1	Cardiomyocyte hypertrophy	Anti-hypertrophic	(108)
	Cardiomyocyte apoptosis	Pro-apoptosis	(126)
miR-15a	Cardiomyocyte apoptosis	Pro-apoptosis	(128)
miR-15b	Cardiomyocyte apoptosis	Pro-apoptosis	(128)
miR-21	Cardiac fibrosis	Profibrotic	(130)
miR-22	Cardiomyocyte hypertrophy	Pro-hypertrophic	(115)
miR-23a	Cardiomyocyte hypertrophy	Pro-hypertrophic	(117)
miR-24	Cardiac fibrosis	Antifibrotic	(133)
miR-29	Cardiac fibrosis	Antifibrotic	(135)
miR-30	Cardiomyocyte apoptosis	Anti-apoptosis	(123)
	Cardiac fibrosis	Antifibrotic	(136)
miR-130	Cardiac fibrosis	Antifibrotic	(136)
miR-132	Cardiomyocyte hypertrophy	Pro-hypertrophic	(118)
miR-133a	Cardiomyocyte hypertrophy	Anti-hypertrophic	(112)
miR-199a	Cardiomyocyte apoptosis	Anti-apoptosis	(125)
miR-208a	Cardiomyocyte hypertrophy	Pro-hypertrophic	(119)
miR-212	Cardiomyocyte hypertrophy	Pro-hypertrophic	(118)
miR-320	Cardiomyocyte apoptosis	Pro-apoptosis	(127)
miR-378	Cardiomyocyte hypertrophy	Anti-hypertrophic	(114)

In this paragraph, we discuss the role of miRNAs in cardiomyocyte hypertrophy, cardiac cells apoptosis, cardiac fibrosis, and circulating miRNAs.

### miRNAs in Cardiomyocytes Hypertrophy

Initially, Zhao et al. reported that miR-1 targets are genes involved in cardiac muscle differentiation and their excessive expression leads to decreased cardiomyocytes proliferation (107). In a mouse model of aortic constriction-induced hypertrophy, cardiac miRNAs expression profile obtained by microarray RNA analysis showed that miR-1 was downregulated since the early phase after cardiac hypertrophy induction. Moreover, *in vitro* overexpression of miR-1 reduced the levels of growth-related factors as fibronectin, Ras homolog enriched in brain, GTPase-activating protein, and cyclin-dependent kinase-9 (108). miR-1 action has been reported to be mediated by calmodulin, calcineurin, and nuclear factor of activating T cells (NFAT) (109).

Activation of insulin growth factor-1 receptor (IGF-R1), P13K/Akt pathway, and inhibition of anti-hypertrophic transcriptional factor FOXO have been referred as the principal mechanisms involved in cardiac hypertrophy induced by miR-1 inhibition (110). Similarly, cardiac samples from patients with hypertrophic cardiomyopathy or mitral stenosis were characterized by decreased miR-1 expression (111).

Interestingly, suppression of miR-133 was associated with a more pronounced cardiac hypertrophy (112). In addition to the main calcineurin/NFAT mechanism of action, miR-133 seems to act through the inhibition of α1-adrenergic receptor (113). Several *in vivo* experimental models of cardiac hypertrophy (pressure overload, volume overload or isoproterenol pumps infusion) showed reduced expression of miR-378 (about 40–60%). *In vitro* overexpression of miR-378 prevented the development of cardiac hypertrophy (114). miR-378 downregulates the expression of IGF-R1, PI3K, and Akt. Xu et al. found that in a rat model of cardiac hypertrophy, the most upregulated miRNA was miR-22. Furthermore, this study reported that miR-22 regulates the fetal gene expression (atrial natriuretic peptide and α-myosin heavy chain) and has an important influence in increasing cardiomyocytes size, partly through targeting phosphate and tensin homolog (PTEN) (115). Overexpression of miR-27b in mice may induce cardiac hypertrophy targeting cardiomyocyte peroxisome proliferator-activated receptor-γ (116). Several studies reported that miR-23a, -212, and -132 mediate cardiac hypertrophy *via* FOXO3 downregulation (117, 118). Callis and colleagues found that overexpression of miR-208a induced cardiac hypertrophy, reduced the expression of thyroid hormone-associated protein 1 and myostatin, 2, and importantly influenced electric conduction resulting in arrhythmias in mice (119). Furthermore, miR-208a was reported among miRNAs able to predict time-dependent cardiomyocyte hypertrophy and other characteristics of cardiac remodeling in endomyocardial biopsies from patients with dilated cardiomyopathy after β-blocker treatment (120).

### miRNAs in Cardiac Cells Apoptosis

miR-21 overexpression in neonatal cardiomyocytes provides a protective role against oxidative-cell induced apoptosis, mediated by NF-κB targeting PTEN/AKT pathway (121, 122).

A reduction in miR-30 expression together with an increase in p53 expression was observed in rat cardiomyocytes in response to apoptotic stimulation. Interestingly, miR-30 regulates the activity of dynamin-related protein-1, a mitochondrial fission protein and the consequent suppression of p53 (123). Furthermore, it has been reported that miRNA-30 regulates the β-adrenergic signaling, targeting β1 and β2 adrenoceptors and G protein αi subunit (Giα2) in doxorobucin-induced myocardial injury (124). Suppression of p53 was also reported as an apoptotic cell effect of miR-199a under hypoxic conditions (125). Overexpression of miR-1 increased cardiac apoptosis in ischemia/reperfusion (I/R) mouse model (126). Mir-320 expression was significantly decreased in I/R model. Overexpression of this miRNA promoted cardiomyocytes apoptosis *in vitro* and increased infarct size *in vivo via* heat shock protein 20 downregulation (127).

miR-15a and miR-15b were upregulated in postischemic HF models, with a more pronounced miR-15b implication. Overexpression of miR-15b led to loss of mitochondrial membrane potential, while its inhibition increased expression levels of the Bcl-2 protein, suppressed the release of mitochondrial cytochrome *c* to the cytosol, and decreased the activities of caspase-3 and -9 (128). Another miRNA with anti-apoptotic effect is miR-92a. It has been reported that the beneficial hypoxia/reoxygenation effect of miR-92a inhibition is mediated by Smad7 (129).

### miRNAs in Cardiac Fibrosis

Several miRNAs have been identified as important regulators of CFs functioning. Initially, it was revealed that miR-21 expression is increased in CFs isolated from HF samples, augmenting ERK-MAP kinase activity through inhibition of sprout protein homolog 1 (SPRY1) and increased levels of fibroblasts growth factor (51). *In situ* studies reported that miR-21 expression is specific for the infarcted myocardium where collagen expression is high and CFs are the most present cell type. In addition, mir-21 regulates the activity of metalloprotease 2 (MMP-2) through PTEN pathway. MMP-2 is well known for its role in ECM turnover, enhancing collagen deposition (130). Similarly, in LV biopsies from patients with severe aortic stenosis, miR-21 expression was significantly higher and correlated either with transvalvular mean gradient and collagen expression (131). The overexpression of miR-21 in CF cultures promoted signal transducer and activator of transcription 3 phosphorylation, increased CF proliferation, and profibrotic gene expression (132).

Other experiments performed on MI animal models identified miR-24 as a key regulator of postischemic HF. Importantly, miR-24 overexpression reduced levels of TGFβ, resulting in modulation of CFs proliferation and migratory properties (133).

In different experimental models of HF, miR-29 expression was highly downregulated within the infarcted area. Modulation of the expression of miRNA-29 regulates collagen production in CFs cell culture (134). CFs behavior in HF is also characterized by their transdifferentiation to a myofibroblast phenotype with enhanced migratory and proliferatory properties. Indeed, it has been reported that miR-29 is able to inhibit the fibrogenic differentiation of fibroblasts mediated by TGF-beta (TGF-β)–Smad3 signaling (135).

In CFs isolated by human LV hypertrophy and in animal models of HF, expression of miRNA-30 and -133 was inversely correlated to CTGF, a promoter of collagen synthesis by CFs. Overexpression of either miR-30 and -133 resulted in the reduction of CTGF and as expected collagen production (136).

### Circulating miRNAs in Heart Failure

Although circulating miRNAs mainly originate from ECs and hematopoietic cells, circulating miRNAs of myocardial origin reflect changes of myocardial tissue miRNAs (137). Ovchinnikova et al. described that circulating miR-18a-5p, -26b-5p, -27a-3p, -30e-5p, -106a-5p, -199a-3p, -652-3p, and -199a-3p were significantly decreased in patients with acute HF compared to healthy controls or patients affected by chronic HF. Importantly, a further decrease of these miRNA levels within 48 h after the hospital admission for acute HF was associated with an increased risk of 6 months mortality (138). A study conducted in a population of ischemic and non-ischemic dilative cardiomyopathy reported that circulating miR-125b and -497 were significantly decrease in ischemic patients, while miR-142-3b and -29b were increased in non-ischemic patients (139). Qiang and collaborators found that miR-126 and -508-5p may predict CV mortality among HF patients (140). Another study described the role of circulating miRNAs in the differential diagnosis of dyspnea in patients with symptomatic HF and in those with dyspnea of non-HF origin. Only miR-423-5p was significantly increased in patients with dyspnea of cardiac origin (141). A translational pilot study reported that in myocardial areas of dyssynchrony miR-30d expression was increased as a protective mechanism. Furthermore, plasma baseline level of this miRNA is associated with response to resynchronization therapy (142). Left ventricular assist devices (LVAD) implantation is emerging as a crucial treatment for end-stage HF patients, interestingly, appearing to determine a good survival rate in elderly patients (143). LVAD implantation is characterized by significant decrease in pulmonary pressure, reduction of circulation time, and neurohormonal changes. Morley-Smith et al. revealed that decreased miR-483-3p and -1202 myocardial and circulating expression was associated with successful response to LVAD implantation (144).

## miRNAs in Hypertension

Hypertension represents one of the most common diseases in adults and a leading risk factor for CVDs and kidney failure (145). Almost 29% of the worldwide population present high blood pressure (BP), and statistics show that this number is intended to growth reaching 1.5 billion by 2025 (145). The risk of developing high BP increases with age: 90% of not hypertensive 55-year-old subjects will develop this pathology later in life (146). Despite the fact that many drugs and changes in lifestyle have shown to decrease BP, currently just 50% of the hypertensive patients are responsive to treatment (147).

Blood pressure is regulated by an ensemble of cardiac, renal, vascular, and neurohormonal mechanisms (148). The pathogenesis of hypertension is complex, involving ECs and VSMCs dysfunction, altered activation of RAAS and SNS together with increase in oxidative stress and inflammation (149). Several animal and human studies indicate that miRNAs contribute to the regulation of these aforementioned mechanisms, representing a new promising potential target for therapies. Moreover, circulating miRNAs are emerging as effective biomarkers in essential hypertension as discussed more in depth below (150).

The RAAS system has a pivotal role in the pathogenesis of hypertension (148). Its persistent and pathological activation can lead to alteration in BP, cardiac contractility, electrolyte balance, as well as vascular resistance and tone (151). Several studies indicate miRNAs to be intrinsically connected with all the components of this hormonal system (summary in Table 3).

**Table 3:** Summarizing table of microRNAs (miRNAs) involved in hypertension.

Tissue	miRNA	Molecular targets	Functions	Reference
Kidney	miR-181a and miR-663	Renin	Renin–angiotensin–aldosterone system (RAAS) pathways regulation	(152, 153)
Vascular smooth muscle cells (VSMCs)	miR-483-3p	Angiotensin-converting enzyme-1 (ACE-1), ACE-2, AT2R, AGT	RAAS pathways regulation	(158)
VSMCs	miR-143/145	ACE, angiotensin II type I receptor (AT1R)	Modulates VSMCs phenotype (downregulation causes shift to synthetic phenotype)	(154, 155)
Endothelial cells (ECs) and VSMCs	miR-155	AT1R ET-1 Endothelial nitric oxide synthase (eNOS)	Decreases: vascular inflammation, ECs migration and VSMSc proliferation OR Impairs endothelium-dependent vasodilatation	(160, 162, 163)
ECs	miR-122	SLC7A1	Decreases nitric oxide production	(166, 167)
ECs	miR-221/222	eNOS Ets-1	Stimulates vascular inflammation	(162)
Plasma and exosomes	miR-130a, miR-195, and miR-92a	AT1R (miR-92a)	Specific expression profile is associated with hypertension	(176)
Peripheral blood mononuclear cells	miR-143/145, miR-133a, miR-21, miR-1 miR-9 and miR-126	Multiple targets, some involved in VSMCs biology	Specific expression profile is associated with hypertension	(177) (178)

Enhanced renin production, characteristic feature of genetically hypertensive mice (BPH/2J), is mediated at least in part by mechanisms involving miR-181a downregulation (152). Genetic expression profile performed on human hypertensive kidneys identified 11 miRNAs differentially expressed in comparison with normotensive kidneys. Among them, miR-663 and miR181a levels were significantly lower in hypertensive patients and have been shown to directly bind renin mRNA (153). Angiotensin-converting enzyme (ACE) catalyzes the conversion of Angiotensin I (Ang I) in its bioactive form Ang II, the main effector of RAAS. ACE is a target of miR-143/145 cluster. Expression of this cluster was increased by shear stress in ECs *via* activation of AMPK-p53 pathway, leading to a reduction in ACE gene expression (154). Moreover, miRNA143/145 expression was fundamental to maintain the contractile phenotype of VSMCs *in vitro* and a regular BP *in vivo*. Loss of miR-143/145 led to an ACE enzyme overexpression and a shift from contractile to synthetic phenotype of VSMCs, which increased the probability to develop neointimal lesions (155). Synthetic VSMCs are characterized by increased proliferation and migration ability. These cells sustain the vascular remodeling occurring during hypertension (156). Using a model of balloon-induced vascular wall injury in rats, two studies showed that, besides miR-143/145, also miR-21 and mir-221/222 are regulators of VSCMs aberrant proliferation and neointimal hyperplasia (97).

Angiotensin II directly influences ECs and VSCMs growth, phenotype, and migration. In addition, Ang II regulates expression of vasoactive molecules, hormones, ECM proteins in vascular cells, myocytes, and fibroblast (157). Kemp and colleagues identified 17 miRNAs specific regulated by angiotensin II type I receptor (AT1R) activation in VSMCs. Among them, miR-483-3p regulates the expression level of four RAAS components: angiotensinogen, ACE-1, ACE-2, and AT2R (158). Chronic Ang II infusion increased expression levels of miR-132 and miR-212 in rats heart, aortic wall, and kidney, whereas treatment with AT1R blockers decreased their levels in arteries of patients subjected to coronary artery bypass grafting (159). In ECs, Ang II induced expression of ET-1, a target of miR-155 and a key regulator in vascular inflammation and remodeling (160, 161). Overexpression of miR-155 reduced: (i) Ang II-mediated migration and inflammatory activation of ECs (162), (ii) AT1R expression in Ang II-treated hypertrophic cardiomyocytes (161), and (iii) Ang II-induced VSMSc proliferation (163).

Endothelium dysfunction is characterized by an imbalance in the release of vasodilatative and vasoconstrictive factors. Hypertension has been associated with an impairment of production of NO, a vasodilator fundamental for the CV homeostasis (164). Mounting evidence showed that its bioavailability could be regulated by miRNAs. Sun and colleagues demonstrated that miR-155 decreases NO release through direct binding and consequent downregulation of endothelial eNOS transcript in HUVEC cells. The authors observed that inflammatory stimuli like TNFα may increase the level of miR-155, resulting in endothelial dysfunction (165). Another study has identified a correlation between a novel polymorphism on the solute carrier family 7 member 1 (SLC7A1) gene and the onset of essential hypertension (166). The variant allele contains one more potential binding site for miR-122 that significantly decreases the expression of SLC7A1, leading to a decrease l-arginine and NO metabolism and endothelial dysfunction (167). GTP cyclohydrolase 1, essential for the regulation of the eNOS activity, is a target of miR-133, which is induced by oxidative stress but inhibited by statin (168).

Oxidative stress contributes to the pathogenesis of CVD, including hypertension. Elevated ROS can impair vascular, renal, and cardiac functions (169). Increased ROS production and miR-21 upregulation were found in heart, liver, kidney, and aorta of spontaneous hypertensive rats (SHRs) in comparison with Wistar control rats. A human study has shown a positive correlation between circulating miR-21 levels and hypertensive state. However, it has been suggested that miR-21 upregulation is part of a compensatory mechanism aimed to decrease ROS level in mitochondria. Interestingly, delivery of exogenous miR-21 lowered BP in the SHR rats, supporting the evidence that miRNAs could constitute a potential therapeutic alternative for hypertension in a near future (170).

Vascular inflammation, together with oxidative stress, is one of the principal features of the endothelial dysfunction (171). As abovementioned, ET-1 promotes vascular inflammation inducing the expression of Ets-1 transcriptional factor. miR-155 and miR-221/222 were able to reduce Ets-1 levels and its downstream target. Among them, VCAM-1, MCP-1, and FLT-1 are major determinants in leukocytes recruitment (162).

### Circulating miRNA in Hypertension

In the last few years, several studies were conducted to compare circulating miRNA in hypertensive versus normotensive patients in order to find reliable biomarker for this pathology. Different studies were performed using high-throughput methodology, like genome-wide array-based technique (150). Li et al. compared miRNA expression level in 13 hypertensive and 5 control patients. Among 27 miRNAs found differentially expressed between the two experimental groups, 3 were validated by qPCR in a larger cohort. Interestingly, hcmv-miR-UL112, a miRNA encoded by the human cytomegalovirus, was upregulated in hypertensive patients. The authors suggested that interferon regulatory factor 1 (IRF-1), target of hcmv-miR-UL112, could be a potential regulator of BP (172). Further studies are needed to confirm this hypothesis. However, IRF-1 can regulate the expression of AT2R and the production of NO in response to inflammatory insult (173, 174). In a similar approach, Yang and colleagues have screened plasma samples from three independent cohorts of patients finding miR-505 increased in hypertension. *In vitro* experiments conducted in miR-505-overexpressing ECs underlined its anti-angiogenic effect (175). Another study was conducted to identify miRNA signature in peripheral blood and exosomes collected from patients exhibiting metabolic risk of CVDs, as metabolic syndrome, hypercholesterolemia, hypertension, and type 2 diabetes. A peculiar expression profile for miR-130a, -195, and -92a was found to be associated with metabolic syndrome and hypertension. Only miR-92a, whose predicted target is AT1R, was found in exosomes (176).

Kontaraki and colleagues have published two studies aimed to analyze miRNAs expression level in human peripheral blood mononuclear cells (PBMCs) using the same cohort of patients (with hypertension, *n* = 60, controls *n* = 29). miR-143/145, miR-133a, mir-21, and mir-1 had a distinct expression profile in PBMCs collected from hypertensive patients. As mentioned before, some of these miRNAs regulate several aspect of VSMCs biology, providing further evidence of the fundamental role of these molecules in the pathogenesis of essential hypertension (177). In addition, the second study showed a lower expression level for miR-9 and miR-126 in hypertensive patients in comparison to healthy controls (178). Finally, it has been found that CD34^+^ circulating progenitor cells from hypertensive patients are characterized by an increased level of miR-221/-222, already related to inflammation and oxidative stress (179).

## miRNAs in Atherosclerosis

Atherosclerosis is a multistage disease characterized by chronic inflammation of the arterial wall, consisting in the progressive remodeling of the vessel intima caused by lipoprotein retention, immune cells recruitment, and endothelial and VSMCs dysfunction. Over time, the lesion progresses and results in the formation of atherosclerotic plaque, ultimately responsible for vessel occlusion and potential thrombotic events. Several studies in the last few years provided with a new molecular insight in the regulation of atherosclerosis, conferring to miRNAs a pivotal role in the regulation of multiple processes related to the plaque formation, progression, and potential rupture (summary in Table 4) (180).

**Table 4:** Summarizing table of microRNAs (miRNAs) involved in atherosclerosis.

Tissue	miRNA	Molecular targets	Functions	Reference
Liver	miR-122	Hmgrc, FAS, SREBP-1	FA and cholesterol homeostasis Pro-atherogenic	(182–184)
Liver	miR-30c	Microsomal triglyceride transfer protein	Low-density lipoprotein secretion and lipid synthesis Anti-atherogenic	(185)
Liver	miR-128-1, miR-148a, miR-130b, and miR-301b	LDL receptor and ABCA1	Lipid metabolism and trafficking Pro-atherogenic	(187)
Liver and macrophages	miR-33	ABCA1	High-density lipoprotein–cholesterol levels regulation Macrophages polarization pro-atherogenic	(188, 189) (203)
Endothelial cells (ECs)	miR-10a	HOXA1 MAP3K7	Anti-atherogenic	(191)
ECs	miR-92a	Itga5 (Sirt)-1, KFL4-2	Pro-atherogenic	(193, 194)
ECs	miR-181b	Importin-α3	Suppression of NF-κB signaling Anti-inflammatory	(195, 196)
Macrophages	miR-27a/b miR-26 miR-302	ABCA1	Cholesterol metabolism (foam cell formation)	(194, 200, 201)

Cholesterol is an essential component of the cell membrane and a precursor of steroid hormones and bile acids. Deregulation of cellular and systemic cholesterol levels predisposes to CV and metabolic diseases.

In the circulation, cholesterol and triglyceride are carried out by lipoprotein complex: low-density lipoproteins (LDLs) deliver cholesterol to peripheral tissues, whereas HDLs transport cholesterol mostly to the liver or steroidogenic organs, where it is metabolized or disposed. Retention of oxidized LDLs (oxLDLs) in the subendothelial space drives the accumulation of macrophages that became inflammatory foam cells. High circulating LDL levels, together with low circulating HDL, are considered a risk factor of atherosclerosis and metabolic disease; miRNAs are able to regulate LDL and HDL biogenesis, representing a target for future treatment (181).

miR-122 is implicated in the metabolism of hepatic cholesterol and fatty acids: *in vivo* studies have shown that the miR-122 inhibition resulted in decreased total cholesterol plasma levels both in mice and non-human primates (182–184).

Microsomal triglyceride transfer protein (MTP) is responsible for the lipidation of apolipoprotein B with consequent LDL production. miR-30c directly binds MTP transcript. Hepatic overexpression of this miRNA decreased LDL secretion and hyperlipidemia in high fat diet mice and led to reduction of atherosclerotic lesions in ApoE^−/−^ mice (185).

In a recent genome-wide association study, almost 200,000 individuals were screened for single-nucleotide polymorphisms (SNPs) associated with abnormal plasma lipid levels. The analysis identified 69 miRNAs localized in proximity of SNPs loci already correlated with dyslipidemia. Among them, miR-128-1, miR-148a, miR-130b, and miR-301b regulate the expression of several proteins involved in lipid metabolism and trafficking, such as LDL receptor (LDLR), whose activity in the liver is responsible for the reuptake and disposal of LDL, and ABCA1, a transporter accountable for formation and secretion of HDL (186). Inhibition of miR-148a and miR-128-1 produced (i) increased levels of expression of LDLR/ABCA1 and augmented LDL clearance in C57BL/6J mice and (ii) modulated levels of circulating lipoproteins in ApoE^−/−^ mice fed with western diet (187).

ABCA1 has been identified as a target of multiple miRNAs. Rayner and colleagues studied the effect of miR-33 downregulation on lipid metabolism and atherosclerosis. miR-33 inhibition increased circulating HDL-cholesterol levels and hepatic ABCA1 expression in both mice and monkeys (188, 189). In addition, mice deficient for the LDLR treated with anti-miR-33 showed enhanced reverse cholesterol transport in the plasma, liver, and feces and a reduction in the atherosclerotic lesion size as well as in lipid content. Interestingly, anti-miR-33 was able to enter also in the plaque macrophages, where it caused a reduction of inflammatory gene expression and increased reparative M2 phenotype (188).

Notably, inhibition of miR-33a/b in monkeys resulted in a marked suppression of the plasma levels of very low-density lipoprotein-associated triglycerides, probably caused by the fatty acid synthesis downregulation alongside upregulation of fatty acid-oxidation genes (189).

Arterial walls are constantly exposed to hemodynamic forces. The vascular endothelium is sensible to mechanical stimuli and acts as a transducer, converting stretch and shear stress in biochemical activation of the cells in order to maintain vascular homeostasis (171). The mechanosensitive miRNAs mainly target pathway involved in NO signaling, inflammation, and leukocytes recruitment (190). Among them, the anti-atherogenic miR-10a and miR-143/45 are increased by laminar flow but decreased by disturbed flow (191, 192). In contrast, mirR-92a is downregulated by laminar and upregulated by oscillatory shear stress; *in vivo* studies have shown its expression to be higher in ECs localized in atheroprone area. Moreover, inhibition of mir-92a in LDLR^−/−^ mice reduced inflammation and atherosclerotic lesions (193, 194).

Atherosclerosis is a chronic inflammatory disease and multiple studies focused on this characteristic. Emerging evidence has identified miR-181b as a master regulator of endothelial inflammation. Overexpression of this miRNA was able to decrease nuclear NF-κB activation exclusively in ECs, suppressing leukocytes recruitment at the atherosclerotic plaque in ApoE^−/−^ mice. Notably, miR-181b expression is downregulated following inflammatory stimuli, both *in vitro* (195, 196) and in plasma of patients affected by CAD, suggesting that miR-181b loss may predispose to development of atherosclerosis (197).

Pro-inflammatory cytokines, such as TNF-α and interleukin1-β, also promote the expression of miR-146a, an anti-inflammatory miRNA widely expressed in immune cells. Recently Ma and colleagues have developed a protocol to deliver miR-146a and miR-181b with an E-selectin-targeting multistage vector directly to the atherogenic lesion. These nanoparticles were able to downregulate chemokines’ expression (CCL2, CCL5, CCL8, and CXCL9) and to reduce macrophage recruitment and atherosclerotic plaque size in male fed a Western diet apoE^−/−^ mice (198).

Several miRNAs are involved in the regulation of macrophage inflammatory activity and phenotype, which is fundamental in the onset and development of vascular lesions. After adhesion to dysfunctional endothelium, monocytes differentiate in macrophages, which play a central role in the lipoprotein uptake in the growing plaque, resulting in the creation of a foam cell layer. Moreover, macrophages promote a chronic inflammatory response secreting a wide range of chemokines and cytokines that stimulate the recruitment of other immune cells. Macrophages homeostasis in the plaque is also regulated by miR-27a/b, targeting genes involved in cholesterol metabolism, like the previously discussed ABCA1 (199). Several other miRNAs, including miR-302 and miR-26, have shown to increase the formation of foam cells inhibiting ABCA1 expression (200, 201).

Macrophages can be classified as M1 (classically activated) or M2 (alternatively activated): the first type presents the classical pro-inflammatory phenotype, while the latter exhibits an anti-inflammatory profile. Atherosclerosis lesions are characterized by the predominance of M1 macrophages (202). As previously mentioned, miR-33 is involved in regulation of lipid metabolism, cholesterol efflux, and inflammatory genes. In addition, miR-33-mediated imbalance between aerobic glycolysis and mitochondrial oxidative phosphorylation induced M1 macrophage polarization. Moreover, inhibition of miR-33 expression in hypercholesterolemic mice resulted in accumulation of M2 macrophages in the plaque (203).

In addition to macrophages, ECs and VSMCs take part in the pathogenesis of atherosclerosis. ECs and VSCMs dysfunction has been already extensively discussed in the previous paragraph regarding miRNAs in hypertension. In addition, VSMCs are able to uptake lipids and transform into macrophage-like foam cells, contributing to the progression of the plaque. Recently, Gabunia and colleagues have identified miR-133 to be a regulator of VSMC foam cell formation, reducing their proliferation and uptake of oxLDLs. Thus, miR-133 may represent a therapeutic target for vascular inflammatory diseases (204).

Atherosclerosis causes clinical manifestation through vessel occlusion or following plaque rupture and thrombotic events. Plaque stability is determined by thickness and composition of the fibrous cap. miR-145 overexpression induced collagen type I and III expression in human aortic VSMCs and promoted the acquisition of stable plaque phenotype (205).

## miRNA in Atrial Fibrillation

Atrial fibrillation represents the most common cardiac conduction disorder, affecting up 2% of the population worldwide (206). CV aging, upon which several risk factors may occur, contributes to the development of the arrhythmia, thus its prevalence is age-related: 70% of affected patients are over 65 years old (207). Constituting a documented cause of stroke, AF is an important contributor to CV mortality and morbidity in elderly (208). Importantly, miRNAs have been shown to play an important role in AF initiation and maintenance.

Electrical and structural remodeling constitutes the main pathophysiological mechanisms responsible for development of AF, which is initiated and maintained by focal ectopic firing, re-entry circuits, or both conditions simultaneously (209). Furthermore, abnormal Ca^2+^ dynamics and neurohormonal dysregulation take part in the making of the conduction disorder (210).

Atrial electrical remodeling is mainly caused by alteration in ionic currents and modification in ionic channels expression and activity. Particularly, the most frequently observed alterations regard increases in inward rectifier K^+^ current (I_K1_) and reduction in inward L-type Ca^2+^ current (I_CaL_) (210). A shortening of the atrial effective refractory period (ERP), consequent to reduction of the action potential duration, and a loss of rate adaptation lead to arrhythmogenesis and AF (211). Several miRNAs are implicated in electrical remodeling (Table 5).

**Table 5:** microRNAs (miRNAs) implicated in atrial fibrillation (AF) electrical remodeling.

miRNA	Target	Model	Findings	Reference
miR-1	KCNJ2 KCNJ2 KCNE1, KCNB2 PP2A Regulatory sub. B56	Normal or infarcted rats Patients with AF Electrically tachypaced rabbits Rat ventricular myocytes	miR-1 overexpression exacerbates arrhythmogenesis miR-1 reduction generates increase in I_K1_ protein Kir2.1 miR-1 upregulation produces reduction of the atrial effective refractory period miR-1 overepression enhances Ca^2+^ release enhances RyR2 activity	(212) (209) (213) (215)
miR-328	CACNA1C, CACNB1	Tachypaced dogs, miR-328 transgenic mice	miR-328 overexpression is related to increased vulnerability to AF due to reduction in L-type Ca^2+^ channel density	(217)
miR-499	KCNN3	Permanent AF patients	miR-499, upregulated in AF, decreases small-conductance calcium-activated potassium channel 3 (SK3)	(218)
miR-26	KCNJ2	Patients with AF, canine, and mice AF models	miR-26 knockdown, inhibition, or mutation enhance Kir2.1 expression	(219)

Yang and collaborators demonstrated miR-1 to be overexpressed in myocardial tissue of CAD patients; its overexpression, in normal or infarcted rat hearts, has shown to exacerbate arrhythmogenesis. Furthermore, miR-1 inhibition, with specific antisense, suppressed the arrhythmogenic events in the infarcted rats (212).

Nevertheless, miR-1 represses GJA1 (encoding connexin 43, the gap junction α1 protein) and KCNJ2 (encoding I_K1_ protein Kir2.1), thus its downregulation results in increase in protein Kir 2.1 expression, a key factor in AF maintenance (209). In electrically tachypaced rabbits, miR-1 upregulation produced a reduction of the atrial ERP (213). Lu et al. reported the reduction of intracellular Ca^2+^ concentration, induced by miR-1, with protective effect against AF (214). Furthermore Terentyev and collaborators noticed an improvement in Ca^2+^ handling related to miR-1 downregulation (215). All the reported evidences support a key role for miR-1 in cardiac electrical activity regulation.

miR-328, which is related to reduction in L-type Ca^2+^ channel density, is upregulated in AF, contributing to atrial electrical remodeling (216). The pro-arrhythmogenic effect of miR-328 overexpression was confirmed in dogs and mice models. Delivery of antagomiR versus this miRNA was able to revert the arrhythmogenic phenotype (217).

Ling and collaborators have proposed atrial miR-499 as a further electrical remodeling promoter, due to downregulation effect on the KCNN3 gene, which encodes the small-conductance calcium-activated potassium channel 3 (SK3). The authors found miR-499 to be highly upregulated in AF (218).

Luo and colleagues underlined the role of miR-26 in the development of AF, reporting the reduced expression of miR-26 isoforms in atrial samples of AF animals and patients, and the consequent upregulation of IK1/KIR2.1. Particularly, adenovirus-mediated expression of miR-26 was related to lower AF vulnerability, while opposite effect was produced by endogenous miR-26 knockdown in mice (219). This evidence suggests the miR-26 to play a protective role in AF. Plasma concentrations of miR-409-3p (a cell proliferator regulator) and miR-432 (targeting myozenin1) were lower in AF patients than healthy ones; Liu and collaborators interestingly noticed that catheter ablation of the conduction disorder may restore their levels (220). Similarly, in AF individuals, the miRhythm study documented a reduction of miR-21and an increase of miR-150 after ablation; both miRNAs are involved in the regulation of atrial remodeling-related genes (221).

## miRNAs in Diabetes Mellitus

Diabetes mellitus affects over 400 millions of adults worldwide and this number is expected to rise up to more than 550 million people by 2030 (222). DM complications are more frequent in elderlies compared to their young counterparts (223). While type 1 diabetes is considered an autoimmune disorder characterized by the destruction of the insulin-producing β cells in the pancreas by the immune system, type 2 diabetes in a multifactorial disease, where genetic predisposition and environmental/lifestyle factors result in decreased insulin sensitivity in skeletal muscles, liver, and adipose tissue and a progressive reduction in pancreatic insulin secretion (224). In both cases, micro- and macro-vascular complications represent a major cause of disability and death. The former includes diabetic nephropathy, neuropathy, and retinopathy, while the latter refers to the increased risk of ischemic heart disease, stroke, and peripheral arterial disease.

microRNAs have been implicated in the pathogenesis of DM and they also represent an attractive biomarker in the diagnosis, prognosis, and response to treatment in this disease (225).

In 2004, miR-375 was identified as a pancreatic islet-specific miRNA regulating insulin secretion, through its target myotrophin (Mtpn), a transcription activator of NF-κB; moreover, the inhibition of miR-375 function resulted in enhanced insulin secretion (226). In further studies on insulinoma cells and primary rat islets, miR-375 was found to directly target 3′-phosphoinositide-dependent protein kinase-1, a key molecule in PI 3-kinase signaling in pancreatic β-cells, suggesting that miR-375 is involved in glucose regulation of insulin gene expression and β-cell growth (227). Furthermore, in mice lacking miR-375, pancreatic β-cells are decreased, while genetic deletion of miR-375 from obese mice (ob/ob), reduced the proliferative capacity of the endocrine pancreas and resulted in a severely diabetic state. Bioinformatic analysis has also shown that miR-375 regulates genes involved in cellular growth and proliferation (228). Moreover, it seems that antagonizing miR-375 may enhance the effects of exendin-4 in DM patients (229).

Another target of miR-124 is Mtpn, which was found to be overexpressed in DM human pancreatic islets, resulting in an impaired glucose-induced insulin secretion (230). Moreover, miR-124a silencing was associated with increased expression of predicted target genes (as Mtpn) involved in β-cell function, suggesting that an altered miR-124a expression may contribute to β-cell alteration (231).

In the regulation of insulin secretion, a role seems to be played by miR-29a and -29b through the silencing of monocarboxylate transporter in β-cells (232); moreover, this family of miRNAs is implicated in the development of insulin resistance in myocytes, through the repression of the insulin receptor substrate 1 (IRS-1), a key molecule in the insulin signaling (233). Other indirect regulators of IRS-1 are miR-103 and -107, whose expression is upregulated in obese mice. Silencing of miR-103/107 leads to improved glucose homeostasis and insulin sensitivity, whereas gain of miR-103/107 function in liver or adipose tissue impairs glucose homeostasis (234). The target of miR-103/107 is caveolin-1, which is a stabilizer of the interaction between IRS-1 and caveolae (235). Several other miRNAs are involved in the pathogenesis of DM, affecting pancreatic β-cell function and insulin resistance, as miR-143, -122, and the let-7 family but further studies are needed to address their potential as therapeutic targets.

### Circulating miRNAs in Diabetes Mellitus

Circulating miRNAs have been proposed as useful biomarkers in the diagnosis, complications risk, and therapeutic management in DM.

Zampetaki et al. analyzed plasma miRNA levels from the Bruneck study population, finding a loss of endothelial miR-126 and other miRNAs in type 2 DM, even before the clinical manifestation of the disease (236). Another study analyzed the expression of diabetes-related serum miRNAs (miR-9, miR-29a, miR-30d, miR-34a, miR-124a, miR-146a, and miR-375) in patients with newly diagnosed DM, impaired glucose tolerance and healthy controls, demonstrating all seven miRNAs to be significantly upregulated in DM and miR-34 has shown the most significant differences (237). In another study in a Chinese population, plasma miR-126 was significantly reduced in susceptible individuals and DM patients compared to normal individuals, suggesting that it may serve as a potential biomarker for early identification of individuals susceptible to DM (238). More recently, another study has indicated that plasma levels of miR-1249, miR-320b, and miR-572 might help differentiating patients with prediabetes and newly diagnosed type 2 DM (239).

In the evaluation of disease progression, a recent study has demonstrated that nephropatic diabetic progressor patients showed significantly greater serum levels of miR-21, miR-29a, miR-29b, and miR-29c in comparison with non-progressors implying the potential usefulness of those miRNAs in monitoring diabetic nephropathy progression (240). Another recent study has shown that circulating endothelium-enriched miR-126 is associated with CAD in DM patients (241).

Other studies in patients with type 1 DM have suggested serum miR-25 levels to be associated with residual β-cell function and glycemic control during disease progression (242); in another type 1 DM cohort, circulating levels of miR-21 and miR-210 were significantly upregulated in plasma and urine of these patients, while urinary miR-126 levels were significantly lower, compared to controls (243). In PBMCs from patients with type 1 DM, it has been demonstrated that miR-21a and miR-93 levels are downregulated compared to controls (244).

Circulating miRNAs in DM might be valuable biomarkers in the diagnosis and identification of the subgroups of patients at higher risk of complications. However, larger studies are needed to establish their potential.

## Summary and Challenging Aspects

Aging is a complex phenomenon involving different systems and usually associated with several comorbidities. miRNAs are key regulators of CV pathophysiology targeting multiple biological pathways. In our review, we have described specific miRNAs involved or altered in CV aging and age-related pathologies. However, due to an expected publication bias, it is difficult to cite failing studies and make a really balanced evaluation. Indeed, the possibility that miRNA studies with negative results were not published at all cannot be excluded.

In this paragraph, we have summarized the main involved miRNAs. Cardiac miR-21 shows a profibrotic role in both HF and aging heart while miR-22 exercises a pro-hypertrophy effect in HF and accelerates fibroblast migration during cardiac aging (51, 52, 110, 125). Hence, miR-21 and miR-22 seem to be a valuable target for elderly patients with HF. In addition, circulating miR-21 levels are increased in older people as well as in patients with HF, hypertension, diabetes, and cancer (50, 97, 98, 177, 243). miR-22 inhibition was able to rescue post-MI cardiac dysfunction in old mice, but not in young ones (56). miR-29 is not an appealing target in old patients with HF since its overexpression could counteract post-MI remodeling but also otherwise sensitize the aorta to the formation of aneurysms (a common phenomenon during aging) (74, 130). Elderly patients are usually affected by atherosclerosis and hypertension (66). The inhibition of miR-122 could constitute a good option for these patients since miR-122 upregulation leads to atherogenesis and endothelial dysfunction (166, 182, 183). miR-181 is downregulated during aging, HF, CAD, and hypertension, suggesting that miR-181 loss is detrimental for CV system (94, 153, 197).

Several circulating biomarkers are now clinically available for diagnosis, prognosis, or guide for treatment of different age-related CVDs (245–247). Moreover, numerous biomarkers of human aging have been studied but, to date, no one has shown consistency in different settings and populations. Some authors tried to evaluate a combination of different biomarkers in order to take in account age-related modifications in different systems and pathways (e.g., inflammation or endocrine and renal function) (248, 249). Unfortunately, we are far away to identify circulating biomarker patterns that stratify the risk of morbidity and mortality for elderly patients with CV diseases. At this regard, since several circulating miRNAs are altered in CV aging *per se* and in different age-related diseases (see above), we strongly believe that a specific combination of few circulating miRNAs would be an effective biosignature panel of CV aging (88, 89). Recently, Navickas et al. systematically reviewed 19 studies and found that miR-1, miR-133a/b, miR-145, miR-208a/b, and miR-499 are the most promising biomarkers for diagnosis and prognosis of CVDs (250).

However, to effectively establish circulating miRNAs as biomarker in clinical practice, developing a standardized method for blood collection as well as miRNAs extraction, detection, and normalization is essential (251, 252). In fact, it is crucial to set the extraction protocol and the storage of blood samples (252–254). Reverse transcription quantitative real-time PCR is the most extensively used method for miRNA profiling while it is still unclear what is the best strategy for data normalization (255–257). Since numerous studies have been performing on this area, we are confident that researchers will be able to standardize the analysis of circulating miRNAs, in a near future (258, 259). Importantly, an internationally validated method will decrease currently elevated costs, allow routinely miRNA identification in research/hospital lab, and regulate ethical aspects.

## Applications and Future Directions

*In vitro* and animal as well as clinical sample-based studies on miRNAs have been helping to understand the development and the progression of age-related CVDs. The potential of miRNAs is evidenced by the increased number of patent applications submitted in the last years (260, 261). miRNA-based treatment is a multi-target therapy resulting in simultaneous regulation of crucial pathways and making it an excellent candidate to modulate complex networks. However, the challenge (at least in part already solved) consists in potential off-target effects, delivery system issues, and safety. Interesting data have been recently published showing the safety and efficacy of miRNA-based therapies in clinical trials. Mimic-therapy for miR-29b, a miRNA involved in fibrous scar formation in the skin, is ongoing phase 1 clinical study in order to evaluate safety and tolerability in healthy volunteers subjected to intradermal injection of the drug.^1^ Importantly, the first antagomiR (miravirsen, anti-miR-122) has been successfully tested in a phase-II trial for the treatment of patients with hepatitis C virus infection (262). Data indicated that miravirsen (as four-week monotherapy) offers long-term suppression of viremia and shows both safety and efficacy. Other clinical trials using antagomiR are ongoing: anti-miR-155 in patients suffering from cutaneous T-cell lymphoma (see text footnote 1) and anti-miR-103/107 for the treatment of non-alcoholic steatohepatitis in patients with type 2 diabetes/prediabetes.^2^

Although miRNA-based therapy to treat CVDs is still in the preclinical phase (small- and large-animal models), the promising advancements in other areas support the great enthusiasm for testing miRNA therapeutics as a new class of drugs for age-related CVDs in the next future. RG-012, a single-stranded, chemically modified oligonucleotide that binds to and inhibits the function of miR-21 has currently been tested in phase I clinical trial for Alport syndrome (see text footnote 2). Since the well-established profibrotic role of miR-21 in cardiac tissue, RG-012 could represent a potential therapy for aged heart. miR-34a, involved in both cardiac and vascular aging, is another promising target to design therapeutics to fight CV aging (58, 62, 68, 263). It has been shown that the inhibition of three miR-34 family members (miR-34a, -34b, and -34c) attenuates cardiac dysfunction in mouse models of HF (92). Furthermore, miR-34c is elevated in the hippocampus of patient as well as mouse models of Alzheimer disease so it has been identified as a potential therapeutic target (91). Taken together, these results suggest the intriguing possibility to inhibit the miR34 family members in elderly patients suffering dementia and HF.

Latest technological improvements in terms of miRNA bioavailability and stability are definitely encouraging, though the tissue specificity and off-target side effects still remain issues to work on. However, the development of effective delivery systems, further studies on large-animal models and clinical trials are required to demonstrate the real application of miRNA-based treatments for age-related CVDs in clinical practice.

One of the major challenges in geriatric medicine is to find reliable biomarkers for diagnosis, prognosis, or prediction of the response to specific drugs. miRNAs represent a very promising tool due to their stability in the circulation and unique signature in CVDs. Detection of multiple miRNAs appears to improve the predictive power compared to single miRNAs.

Despite several publications identified miRNA as a remarkable target for diagnosis and treatment of age-related CVDs, further studies are needed to investigate their translational potential in the real clinical practice.

## Author Contributions

All authors participated in writing the manuscript and/or revising it critically for important intellectual content.

## Conflict of Interest Statement

The authors declare that the research was conducted in the absence of any commercial or financial relationships that could be construed as a potential conflict of interest.

## Footnotes

^1^http://www.miragentherapeutics.com/clinical-trials/.

^2^www.Regulusrx.com.
